# Tumor antigen CA125 suppresses antibody-dependent cellular cytotoxicity (ADCC) via direct antibody binding and suppressed Fc-γ receptor engagement

**DOI:** 10.18632/oncotarget.19090

**Published:** 2017-07-07

**Authors:** James Bradford Kline, Rina P. Kennedy, Earl Albone, Qimin Chao, Shawn Fernando, Jennifer M. McDonough, Katherine Rybinski, Wenquan Wang, Elizabeth B. Somers, Charles Schweizer, Luigi Grasso, Nicholas C. Nicolaides

**Affiliations:** ^1^ Morphotek Inc., Exton, PA, USA

**Keywords:** CA125, ADCC, farletuzumab, Fc-γ receptor, ovarian cancer

## Abstract

Cancers employ a number of mechanisms to evade host immune responses. Here we report the effects of tumor-shed antigen CA125/MUC16 on suppressing IgG1-mediated antibody-dependent cellular cytotoxicity (ADCC). This evidence stems from prespecified subgroup analysis of a Phase 3 clinical trial testing farletuzumab, a monoclonal antibody to folate receptor alpha, plus standard-of-care carboplatin-taxane chemotherapy in patients with recurrent platinum-sensitive ovarian cancer. Patients with low serum CA125 levels treated with farletuzumab demonstrated improvements in progression free survival (HR 0.49, *p* = 0.0028) and overall survival (HR 0.44, *p* = 0.0108) as compared to placebo. Farletuzumab’s pharmacologic activity is mediated in part through ADCC. Here we show that CA125 inhibits ADCC by directly binding to farletuzumab that in turn perturbs Fc-γ receptor engagement on effector cells.

## INTRODUCTION

Human cancers employ a number of mechanisms to evade host immune responses against novel antigens generated from aberrant over-expression, mutations and/or epigenetic alterations [1]. Recent studies have found that tumors utilize naturally occurring immune checkpoint pathways in order to avert host immune responses. While these pathways are now being exploited to generate novel therapies, clinical responses have varied widely suggesting that additional mechanisms of immune resistance exist. This view has been supported by the recently concluded 1100 patient MORAb-003-004 (NCT00849667) Phase 3 clinical trial in patients with first-relapsed, platinum-sensitive ovarian cancer. The trial tested the experimental agent farletuzumab plus standard-of-care (SOC) (carboplatin plus taxane) as compared to placebo plus SOC in a randomized, double-blinded design [2]. While the overall study did not statistically meet its primary endpoint, prespecified subgroup analysis identified a responding subpopulation. Patients with low serum CA125 levels [no greater than 3X the upper limit of normal (< 3X ULN)] treated with 2.5 mg/kg farletuzumab demonstrated improvements in both progression free survival (PFS) (hazard ratio [HR] 0.49, *p* = 0.0028) and overall survival (OS) (HR 0.44, *p* = 0.0108) as compared to patients treated with SOC and placebo. The effect did not appear to be prognostic as the placebo control group (*N* = 357) showed no difference in response in relation to low vs. high serum CA125 levels [PFS (HR 0.88, *p* = 0.481) and OS (HR 0.90, *p* = 0.638)].

Farletuzumab is a humanized IgG1 monoclonal antibody (mAb) targeting folate receptor alpha (FRA) whose mode of action includes antibody dependent cellular cytotoxicity (ADCC) [3]. While the antibody has been shown to have additional anti-tumor properties including autophagy-induced cell killing [4], its ADCC activity has been shown to be critical for its anti-tumor effects *in vivo* [5]. This was demonstrated in human ovarian cancer xenografts whereby farletuzumab anti-tumor activity was completely abolished when amino acid residues in the Fc receptor binding domain were modified. As part of the MORAb-003-004 Phase 3 clinical study, prespecified subgroup analysis included investigating outcome in patients with soluble serum CA125 (sCA125) levels above or below the cut-point of 3X ULN. This was based on prior clinical studies that suggested certain sCA125 threshold levels may indicate level of disease bulk [6] as well as reports that sCA125 may have immuno-suppressive effects on ADCC [7]. Biomolecular studies have shown that a strong correlation exists between serum CA125 and cell surface tissue expression in epithelial ovarian carcinomas (EOC) [8, 9]. Patients with serum CA125 levels greater than 100 U/mL were found to have tumor tissue expression exceeding 10,000 U/mL [10]. Moreover, diagnostic studies measuring interpatient serum and soluble tissue CA125 levels have also found that local CA125 levels exceed corresponding serum values by more than 2,000-fold [11]. While these data suggest that high serum CA125 levels likely correlate with significantly higher levels of either sCA125 and/or membrane-bound CA125 (mCA125) within EOC lesions, the correlation between tumor lesion size and sCA125 levels has been equivocal to date, likely due to the small sample sizes tested as well as disease heterogeneity.

CA125/MUC16 is the largest protein from the mucin family with a predicted molecular weight of 2353 kDa, which is increased by as much as 2-fold by hyperglycosylation of its extracellular domain. It is over-expressed on tumor cell membranes in most EOC and in subsets of other cancer types (i.e. mesothelioma, lung, pancreatic and breast carcinoma) [12, 13]. sCA125 derives from cleavage of the mCA125 by matrix metalloproteinases and neutrophil elastases leading to its presence in systemic circulation and accumulation in peritoneal fluid [14]. There is some evidence that both sCA125 and mCA125 can inhibit immune-effector activities of lymphocytes, in particular ADCC activity. While its mechanism on immune-effector suppression is unknown, previous studies have shown that sCA125 could suppress NK-mediated ADCC by binding to Siglec-type cell surface receptors and causing downregulation of Fc activating receptors [7]. mCA125 has also been reported to have immuno-suppressive effects on NK cell-mediated ADCC [15]. Most recently, it has been shown that sCA125 can specifically bind to antibodies with mid to low nanomolar affinities [16].

Based on the clinical outcome in low sCA125 patients in the MORAb-003-004 Phase 3 clinical trial [2] and reported immuno-suppressive effects of CA125 on ADCC, it was suggestive that these effects may be a result of CA125 biological activity on patients’ immune cells. To explore this hypothesis, additional *post hoc* analyses of clinical specimens from the Phase 3 study were conducted. These included clinical correlations of CA125 and other commonly monitored tumor-shed antigens (TSA) on clinical response, correlation between interpatient sCA125 levels and tumor lesion bulk as well as other clinical and biomolecular variables. Here we report the findings that sCA125 levels, unlike other TSAs or biomarkers tested, are predictive of farletuzumab's clinical response. This correlation can be explained by functional studies that unveil a negative impact of CA125 on IgG1-mediated ADCC via its direct binding to a subset of antibodies and perturbation of antibody-CD16a Fc-γ receptor engagement. The finding that CA125-antibody interaction occurs across a subset of other mAbs suggests that CA125 tumor production may represent an immuno-suppressive mechanism to aid tumors ability to avoid host immune responses.

## RESULTS

### Identification of the tumor-shed antigen CA125 as a predictor of farletuzumab immune-mediated response in patients with relapsed platinum-sensitive ovarian cancer

**Figure 1:**
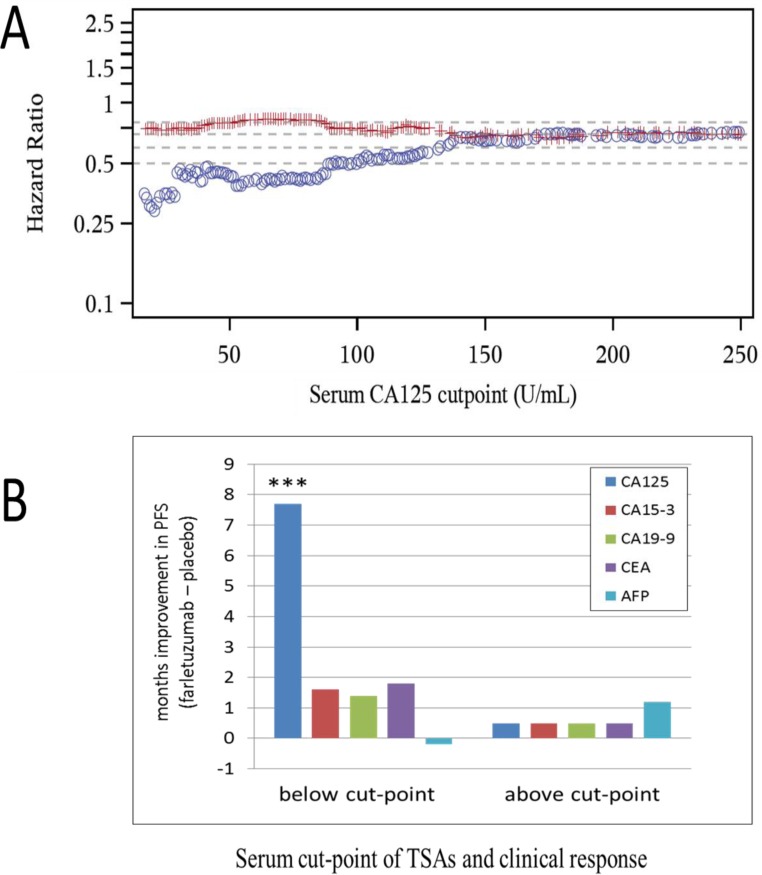
Clinical response of patients treated with farletuzumab at varying baseline levels of CA125 and other TSAs **A**. PFS as determined by Hazard Ratio (HR) comparing farletuzumab to placebo treatment in *entry set* samples with baseline serum CA125 levels. Blue circles represent patient PFS HR values in those treated with placebo vs farletuzumab with CA125 below the cut-point and red lines represent patient PFS HR treated with placebo vs farletuzumab with CA125 above the cut-point. A significant linear increase in PFS improvement [considered to be a HR value of approximately < 0.5; (18)] is observed in patients treated with farletuzumab when sCA125 levels are less than 4X ULN. **B**. PFS responses (in months) in patients from the *post hoc set* stratified above or below the optimal cut-point for each TSA comparing patient responses in farletuzumab vs placebo treatment group. CA125 was the only TSA to show a predictive and significant improvement in PFS (7.7 months improvement) in patients treated with farletuzumab plus SOC (dark blue bar). These values were in a similar range to those derived from the *entry set*. *** One-sided *p* < 0.0005.

Serum CA125 and tumor measurements by computerized tomography (CT) scan were conducted on all patients eligible for MORAb-003-004 Phase 3 clinical study entry. The sum of the longest diameter (SLD) of target lesions for each patient was measured and documented prior to first treatment to support the primary endpoint of PFS by RECIST v1.0 [17]. Serum analysis of CA125 was conducted on all eligible patients entering the trial (referred to as the *entry set*). A subset of patients (549 out of 1100) consented to having additional biomarker studies conducted *post hoc* (referred to as the *post hoc set*) that could aid in understanding the potential prognosis of disease as well as the potential prediction of therapeutic response to the SOC chemo-backbone in the presence or absence of farletuzumab treatment. The number of samples from placebo and farletuzumab treated arms were well balanced in both test sets. As shown in Figure 1A, a robust and linear improvement in PFS exists from high to low sCA125 in patients from the *entry set*, in which the blue circles represent the HR of comparing PFS in farletuzumab (2.5 mg/kg) to placebo treated patients below each CA125 baseline cut-point value and the red line representing the HR in patients treated with farletuzumab vs placebo with CA125 levels above each cut-point value. This effect appeared to be significant in patients having up to and no greater than 4X ULN of CA125 (ULN for the clinical diagnostic test used in the study is 21 IU/mL). These effects were consistent when a similar analysis was conducted via Kaplan Meier plot of patients exhibiting sCA125 levels less than 1 to 4 X ULN (Supplementary Figure 1). Analysis of other elevated tumor shed antigens (TSA) commonly monitored in other prominent cancer types including CA15-3, CA19-9, CEA and AFP (PSA was omitted due to gender specificity) [19, 20] on clinical response were conducted from the serum of 549 patients in the *post hoc set*. First, optimal cut-point analysis was performed for each TSA and clinical response was measured as described in the methods. CA125 was the only TSA showing statistically significant clinical benefit for PFS (7.7 months improvement) comparing farletuzumab to placebo treated patients with low sCA125 levels within the *post hoc set* (Figure 1B). The majority of the patients receiving farletuzumab in this low CA125 set were still surviving at the time of analysis and the median OS was not estimable, as compared to a median OS of 29.1 months in the patients receiving placebo, although the difference was statistically significant (one-sided *p* = 0.0157). To determine if any other commonly monitored clinical or known molecular variable correlated with a predictive response to farletuzumab treatment, *post hoc* analysis was conducted on 23 additional variables. Statistical analysis found that only serum CA125 baseline levels were predictive of farletuzumab response. Statistical analysis of clinical and other biomolecular covariates in addition to the TSAs described above found that they were either not predictive of farletuzumab response, or were prognostic as they showed similar effects in the patients from the placebo control group. These other covariates have been previously presented in other *post hoc* studies [21–23]. The prognostic factors included lesion size at screening, length of patient's first remission, baseline albumin levels, soluble FRA (sFRA) levels and BRAC status. Multivariate Cox model on the intent-to-treat (ITT) population also confirmed sCA125 as a predictive biomarker adjusting for other commonly monitored clinical prognostic factors.

Previous studies have suggested that serum CA125 levels correlate with disease bulk [6, 24]. Analysis of 1100 patients from the *entry set* with varying sCA125 levels did not demonstrate a significant correlation between tumor lesion size and sCA125 levels at baseline (Pearson correlation coefficient of 0.075) meaning that sCA125 levels do not reflect tumor bulk. These data suggest that tumors may yield high or low levels of sCA125 regardless of lesion size.

Antibodies that utilize ADCC tumor killing may potentially be affected by level of target expression, lack of NK cell tumor penetration as well as polymorphisms in the CD16a Fc-γ receptor that affects antibody binding affinity to CD16a Fc-γ receptor. While we were unable to obtain tumor biopsies of relapsed tumors from patients to directly measure FRA tumor expression levels, sFRA has been found to correlate with cell surface tumor expression levels [25]. *Post hoc* analysis of patients found that while sFRA levels were prognostic, they were not predictive of farletuzumab response in the *entry set* or subgroup population. While NK cells have been previously reported to be present within ovarian tumors [26], *post hoc* analysis of CD16a isoforms V158F in patients from the *post hoc set* found no difference with regards to farletuzumab or placebo subgroup clinical outcome.

### sCA125 inhibits farletuzumab-mediated ADCC

**Figure 2:**
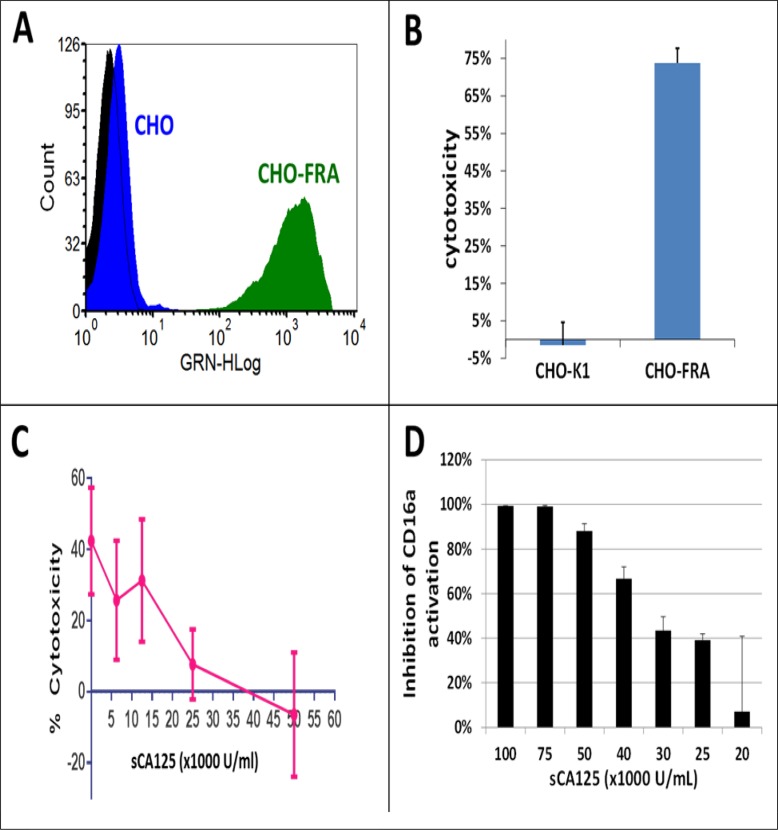
Effects of sCA125 on ADCC **A**. FACS analysis of parental CHO and CHO-FRA cells using farletuzumab demonstrates robust cell surface expression of FRA in CHO-FRA cells. Black peak represents unstained cells; blue and green peaks represent farletuzumab-stained CHO and CHO-FRA cells, respectively. **B**. CHO and CHO-FRA cells were incubated with 10 μg/mL of farletuzumab in the presence of PBMCs. Farletuzumab mediates PBMC ADCC against CHO-FRA but not CHO cells. **C**. Farletuzumab-mediated NK cell ADCC. CHO and CHO-FRA cells were incubated with 10 μg/mL of farletuzumab in the presence of primary NK cells. sCA125 was found to inhibit farletuzumab-mediated NK cell ADCC in a dose dependent manner. **D**. sCA125 effects on Jurkat-Luc cells. Jurkat-Luc cells were incubated with 6 μg/mL of farletuzumab and CHO-FRA cells with increasing concentrations of sCA125. As shown, sCA125 inhibits farletuzumab-mediated ADCC signaling in a dose-dependent manner. All data represent values of at least triplicate experiments and all meet *p* < 0.05.

To test the effects of sCA125 on ADCC mediated by primary human peripheral blood mononuclear cells (PBMCs), we engineered Chinese hamster ovarian (CHO) cell lines to express human FRA (CHO-FRA). ADCC assays employing primary effector cells confirmed the ability of farletuzumab to elicit ADCC against CHO-FRA but not against parental CHO cells (Figure 2A and 2B). Two additional immune-effector systems were used to further test the potential effects of sCA125 on ADCC of CHO-FRA cells. One employed human primary NK cells isolated from PBMCs of normal healthy volunteers and the other employed Jurkat-luciferase (Jurkat-Luc) effector cells, which are engineered to express the human CD16a Fc-gamma receptor (CD16a/FcγRIIIA). This system emits a reporter-based signal upon engagement of Jurkat-Luc cells with target-bound IgG1. Because CD16a activation in primary immune-effector cells precedes the cytotoxic processing steps (reviewed in Supplementary Figure 4), this system aids in narrowing down the potential mechanism by which sCA125 suppresses ADCC activation. As shown in Figure 2C, sCA125 can suppress the ADCC activity of NK cells in a dose-dependent fashion. A similar effect was observed using Jurkat-Luc cells (Figure 2D) suggesting that the immuno-suppressive effect by sCA125 resides somewhere in the process of antibody-CD16a engagement and proximal downstream signaling.

**Figure 3:**
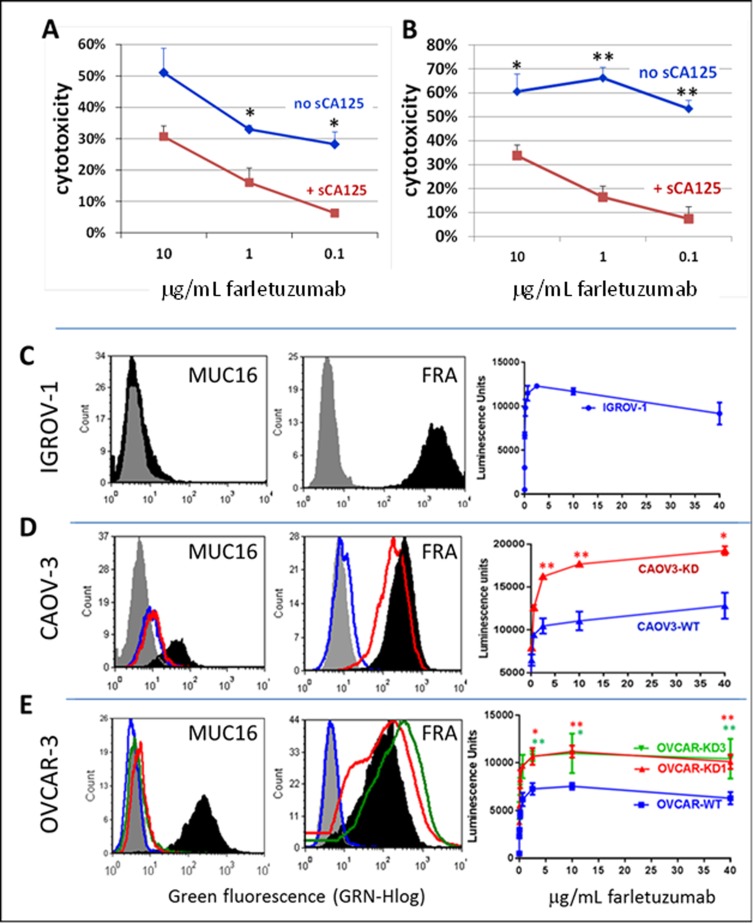
Suppressive effects of sCA125 and mCA125 on farletuzumab-mediated ADCC Soluble CA125 inhibited farletuzumab-mediated ADCC against IGROV-1 **A**. and CAOV-3 **B**. ovarian cancer cells using primary human PBMCs as effector cells. Shown is percent ADCC-mediated cytotoxicity on target cells at different concentrations of farletuzumab and 30 KU/ml sCA125. * *p* < 0.05; **p < 0.01. Figures C-E demonstrate the effects of membrane-bound CA125 on ADCC using Jurkat-Luc reporter cells. IGROV-1 **C**., CAOV-3 **D**. and OVCAR-3 **E**. ovarian cancer cell lines were tested for farletuzumab-mediated ADCC activity. The latter two lines were engineered using optimized shRNA vectors to suppress endogenous CA125/MUC16 expression (shown in left graphs where black is CA125 expression in parental cells and red or green in KD cells). Gray peaks represent parental cells and blue peaks represent KD cells stained with secondary antibody only. Farletuzumab-mediated ADCC activation occurred in all parental lines (right-side graphs, blue lines) and was significantly increased in shRNA-CA125 suppressed CAOV-3 (CAOV3-KD red line) and OVCAR-3 (OVCAR-KD1 red line and KD3 green line) cells when incubated with greater than 2.5mg/mL farletuzumab. * *p* < 0.05; ** *p* < 0.01.

To confirm the ADCC results in Figure 2, we next tested for ADCC against human cancer cells that naturally express FRA and CA125. As seen with CHO-FRA cells, sCA125 could inhibit farletuzumab-mediated ADCC against ovarian cancer derived IGROV-1 cells (Figure 3A), as well as CAOV3 cells (Figure 3B). Overall, these data support the notion that sCA125 has inhibitory activity on farletuzumab-mediated ADCC against FRA expressing cells, and the magnitude of this effect increases with higher concentrations of sCA125 or with lower concentrations of farletuzumab. Similar effects were observed using other mAbs that recognize tumor cell surface markers and whose anti-tumor effect is mediated in part by ADCC (data not shown). In addition, anti-tumor effects by farletuzumab using ovarian cancer xenografts find those that express CA125 are less responsive to tumor growth suppression (personal observations, 4).

### Membrane bound CA125 (mCA125) inhibits farletuzumab-mediated ADCC

mCA125 has been reported to inhibit antibody-independent natural killing [15] but its effect on ADCC was unknown. Recombinant expression of mCA125 in CA125-negative cells has been encumbered by its very large molecular size to produce levels of full length or truncated gene product similar to those in naturally producing tumor cell lines. To evaluate mCA125 effects on ADCC, we employed ovarian cancer cell lines that have inherent high levels of mCA125 and engineered their mCA125-suppressed counterparts via stable shRNA knockdown. Two different isogenic high and low mCA125 expressing ovarian cancer cell lines were generated using independent CA125 shRNA lentiviral knockdown constructs. As shown in Supplementary Figure 2C and 2D, their phenotype and growth rates were similar to their parental counterparts. IGROV-1 served as a control cancer cell line for low CA125 as they are negative for mCA125 and strongly positive for FRA (Figure 3C). Using the Jurkat-Luc reporter assay, there was a dose-dependent activation of effector cells when incubated with up to 40 mg/mL farletuzumab. CA125 shRNA knockdown in CAOV3 cells (CAOV3-KD) resulted in low, if any, mCA125 and maintained FRA expression similar to the parental line (Figure 3D). Employing the Jurkat-Luc system, farletuzumab was able to mediate a 60% increase in ADCC signaling against CAOV3-KD cells as compared to the parental line. No effect on ADCC was observed for either line when using an antibody whose antigen is not expressed by CAOV-3 (Supplementary Figure 2A).

These results were confirmed using two independent CA125 shRNA constructs (KD1 and KD3) in the OVCAR3 human ovarian cancer cell line (Figure 3E). OVCAR3-KD1 and -KD3 both had reduced mCA125 and maintained FRA expression similar to the parent cell line. Farletuzumab was shown to mediate a significant increase in ADCC signaling against both OVCAR3-KD1 and -KD3 cells as compared to parental. No ADCC effect was observed in either line when an irrelevant mAb was used (Supplementary Figure 2B). Similar suppressive effects by mCA125 on ADCC signaling were observed when using an IgG1-type mAb to another cell surface antigen equally expressed between OVCAR and OVCAR-KD1 and -KD3 (data not shown). Based on their similar response profiles, OVCAR-KD1 cells were used in all subsequent experiments.

### Cellular and molecular events potentially involved with CA125-mediated ADCC immunosuppression

**Figure 4:**
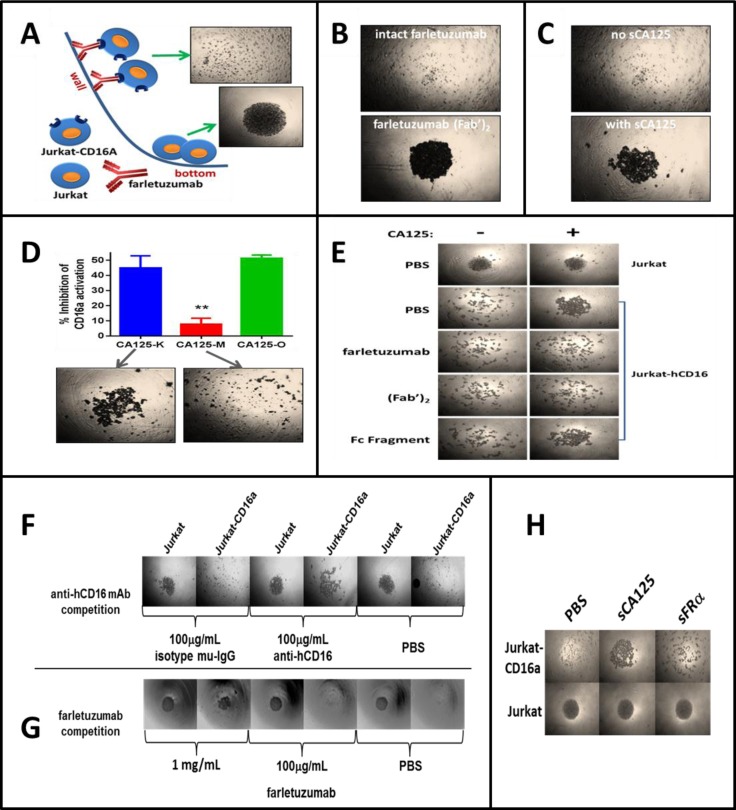
Overview of the BRA assay and effects of sCA125 on CD16a-antibody binding Microwell plates were coated with farletuzumab, farletuzumab fragments or control agents and Jurkat-CD16a or parental cells analyzed for their ability to adhere to the wall surface. Cells that can adhere remain tethered to the well wall while those that cannot roll and cluster at the U bottom. **A**. Jurkat cells roll (bottom panel) in contrast to Jurkat-CD16a (top panel) which are retained via farletuzumab Fc-γ receptor binding. **B**. Wells were coated with farletuzumab or its (Fab’)_2_ and Jurkat-CD16a are tested for rolling. As shown, cells adhere to the full length farletuzumab (top) but not (Fab’)_2_ coated wells (bottom) suggesting farletuzumab's Fc domain is required for the Jurkat-CD16a-farletuzumab tethering. **C**. Jurkat-CD16a or NK cells were incubated in wells coated with farletuzumab and incubated with or without sCA125. sCA125 inhibited Jurkat-CD16a-farletuzumab (bottom panel) as well as NK cell-farletuzumab binding (not shown). **D**. sCA125 was absorbed on CHO-MSLN vs CHO cells and the remaining supernatant was used in ADCC and BRA assays. sCA125-depleted sample buffer no longer inhibited farletuzumab ADCC activity (red bar, top graph) or CD16a-farletuzumab engagement in BRA assay (bottom pictures). CA125-K (blue) is sCA125 absorbed on CHO cells (sCA125^+^); CA125-M (red) is sCA125 absorbed on CHO-MSLN cells (CA125^−^); CA125-O (green) is starting sCA125 preparation (CA125^+^). **E**. Full-length farletuzumab and the (Fab’)_2_ domain are able to compete with sCA125 for Jurkat-CD16a binding to microwells coated with farletuzumab, suggesting that CA125 perturbs CD16a-farletuzumab binding via interaction within the Fab region. Top row is Jurkat parental. All other rows employ Jurkat-CD16a plus competitors listed on left. Competition assays using: **F**. neutralizing anti-CD16 antibody; **G**. excess farletuzumab; or **H**. sFRA show farletuzumab-CD16a Fc-γ receptor binding specificity for the assay.

IgG1-mediated ADCC is a complex process that is regulated by various events and factors. Supplementray Figure 4 illustrates some of the known cellular and molecular events involved in the ADCC process and outlines the 6 key steps leading to cellular cytotoxicity. Since CA125 may be targeting one of these steps, we investigated their possible involvement with CA125-mediated suppression. The ADCC suppression observed on Jurkat-Luc cells (Figures 2 and 3) suggested that CA125 suppression was already evident at the upstream CD16a activation step (Supplementary Figure 4, step 5 and 6). We therefore proceeded to design experimental systems to explore the steps that are upstream of, and include, step 5 (antibody-CD16a binding and CD16a activation).

We first conducted a number of validation experiments to define our system and rule out results due to potential artifact. We demonstrated that sCA125 did not have a cytotoxic effect against effector cells, which could decrease ADCC signal and that sCA125 did not reduce farletuzumab binding to target cells (step 2) (Supplementary Figure 2A and 2B). Step 4 of the ADCC process involves the activity of regulatory receptors. Previously, sCA125 was shown to bind the NK inhibitory receptor Siglec-9 and suppress CD16a function within these cells [27]. However, Jurkat-Luc effector cells were found to express little to no Siglec-9 or the complementary inhibitory receptor Siglec-7 (Supplementary Figure 5A). While parental Jurkat cells lack Siglec-7 or Siglec-9 expression (personal observations, [27]), a subset of NK cells have been found to express Siglec-7 and -9. Therefore, we cannot rule out that these receptors play some role in CA125-mediated immunosuppression, or that other regulatory receptors on the effector cells could interact with sCA125. However, we have been unable to demonstrate direct binding of sCA125 by immunostaining using a CA125-specific antibody or biotinylated-sCA125 on Jurkat-Luc cells, CHO-FRA target cells or IGROV-1 cells suggesting the effects observed in our system are via a different mechanism(s) than inhibitory receptor suppression.

### Role of CD16a in CA125-mediated ADCC suppression

In light of the above results, we hypothesized that CA125 may directly interfere with farletuzumab-mediated CD16a activation. sCA125 has been previously reported to downregulate CD16a surface expression in human NK cells [7] and this effect could potentially disrupt CD16a function. However, while we did observe a slight effect on CD16a expression on CA125 exposed NK cells, we did not observe any change in CD16a expression levels on the ectopically-expressed CD16a in Jurkat-Luc cells treated with sCA125, therefore ruling out decreased CD16a expression as a potential mechanism in our ADCC defined Jurkat-Luc system (Supplementary Figure 6B and 6A, respectively).

**Figure 5:**
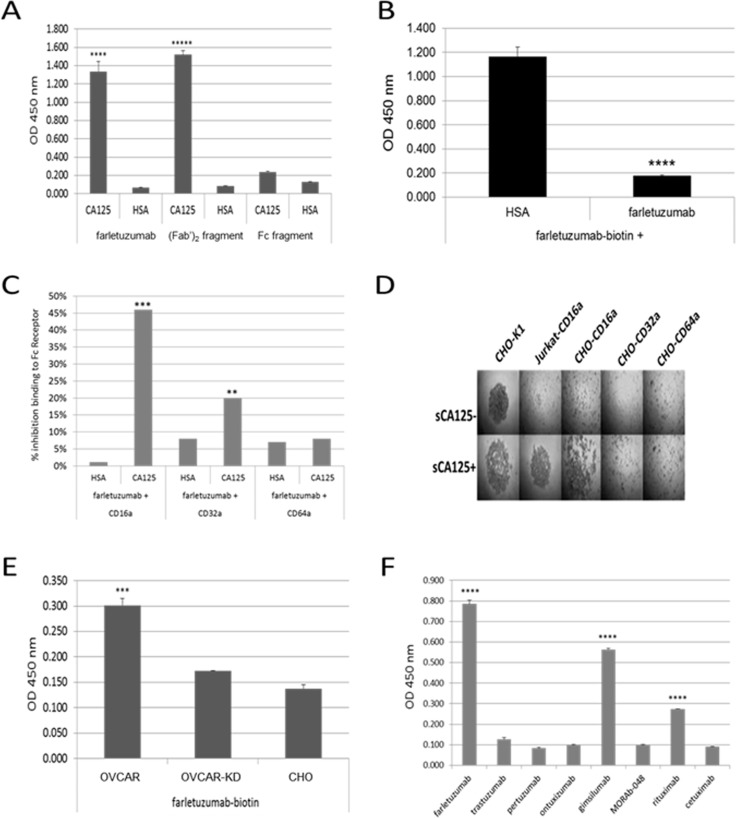
ELISA assays using purified reagents demonstrate CA125 binding to farletuzumab and other independent antibodies that result in reduced antibdy-CD16a and -CD32a Fc-γ receptor engagement **A**. Biotinylated farletuzumab and its (Fab’)_2_ fragment, but not its Fc fragment bind immobilized sCA125. 96-well plates were coated with 15 KU/mL sCA125 or human serum albumin (HSA) and probed with biotin-labeled farletuzumab, (Fab’)_2_ or Fc fragments. **B**. Farletuzumab is able to compete for farletuzumab-biotin binding to sCA125. **C**. sCA125 suppresses Fc receptor binding to farletuzumab. Farletuzumab was incubated alone or with sCA125 or HSA and probed with biotinylated-CD16a, -CD32a or -CD64a Fc receptor and graphed as a percent inhibition of Fc receptor binding compared to control. sCA125 caused a significant decrease of CD16a binding to farletuzumab as compared to controls (46%, *p* < 0.0002). Reduction in farletuzumab binding to CD32a Fc receptors was also significant (12%, *p* < 0.003) while minimal inhibition was observed by sCA125 on farletuzumab binding to CD64a Fc receptor (1%, *p* = 0.772). Similar reactions were probed with anti-human IgG-HRP to confirm incubation of farletuzumab with sCA125 did not result in less farletuzumab binding to wells (Supplementary Figure 8). **D**. BRA assay using CHO cells expressing human Fc activating receptors show only farletuzumab-CD16a interactions are disrupted by sCA125. **E**. Farletuzumab has a significant increase in binding to the mCA125 producing OVCAR parental as compared to mCA125-suppressed isogenic OVCAR-KD1 or mCA125-null CHO membranes. **F**. sCA125 can bind to subsets of other humanized and fully human mAbs. Seven purified mAbs were biotinylated and used to probe wells coated with 15 KU/mL sCA125 or HSA. While all antibodies were able to robuslty and equally bind their respective target antigen, only a subset were able to specifically bind sCA125 as compared to HSA controls. All ELISAs were done in at least triplicate and different lots of sCA125 or cell membrane preparations were used with similar results. In panels A and B *** *p* < 0.0001; **** *p* < 0.00007; ***** *p* < 0.000001, panels E and F *** *p* < 0.0001; **** *p* < 0.00001.

Another possible mechanism of ADCC suppression is that sCA125 may perturb the interaction between CD16a and the farletuzumab Fc domain (Step 3). An essential aspect of this interaction is that multiple CD16a-IgG1 complexes form at the effector-target cell interface resulting in strong cell-cell engagement and CD16a activation [28]. CD16a is unable to bind monomeric IgG due to its low affinity and therefore requires aggregates of antibody Fc to bind and retain engagement via avidity [29]. To recreate this scenario, we designed a system whereby U-bottom microwell plates were coated with farletuzumab to mimic its aggregation on target cell surface and tested whether it could mediate adherence of Jurkat-CD16a cells to the well wall surface as compared to Jurkat (CD16a-null) parental cells. If cells adhered to the well wall, the cell density at the well bottom would be sparse. If cells did not adhere to the well wall then they would roll and form a dense cluster at the well bottom. As shown in Figure 4A, parental Jurkat cells rolled and clustered at the well bottom (bottom picture) while Jurkat-CD16a cells, presumably tethered by multiple farletuzumab-CD16a interactions, adhered to the wall leaving a low density of cells at the bottom of the well (+ picture). To demonstrate that CD16a-farletuzumab Fc engagement was responsible for tethering across the well surface, independent wells were coated with serum albumin (Supplementary Figure 6C) or the farletuzumab (Fab’)_2_ fragment, which lacks the Fc domain (Figure 4B), and Jurkat-CD16a cells were tested for adherence. As shown, neither of these proteins maintained Jurkat-CD16a adherence to the well wall thereby demonstrating that the Fc domain is required for farletuzumab-dependent adhesion of Jurkat-CD16a cells. In addition, a CD16a blocking mAb abrogated Jurkat-CD16a cell adhesion as did competition with a 100-fold molar excess of farletuzumab, further validating this CD16a-farletuzumab binding system (Figure 4F and 5G). By harnessing the specificity of this biological rolling assay (referred to herein as BRA assay), we observed that sCA125 could inhibit farletuzumab-dependent adherence of Jurkat-CD16a as well as NK cells (Figure 5C). In contrast, sCA125 did not inhibit fibronectin-dependent adherence of Jurkat-CD16a cells, which is able to tether both Jurkat and Jurkat-CD16a cells across the well surface (Supplementary Figure 6C), suggesting that inhibition of cell adhesion is not a general property of CA125 but a more specific effect on antibody-Fc-γ receptor binding.

CA125 is a large, molecularly complex glycoprotein that can be isolated from cell lines or patient ascites using purification methods described in the Supplementary Methods. Although SDS-PAGE or FPLC analysis of patient derived sCA125 does not detect low molecular weight contaminants after processing, we utilized multiple methods to rule out any possible effects by minor contaminants that could influence adherence in the BRA assay. We used an optimized CHO-MSLN cell affinity method to deplete sCA125 from our preparations by exploiting the ability of CHO-MSLN cells to absorb sCA125 via high affinity (Supplementary Figures 3C, 3D and 6D) [30]. Using this method, typically 98% of sCA125 is removed from the CA125 sample prep. Depletion of sCA125 from the sample reversed the inhibitory effect on farletuzumab-CD16a ADCC activation and adherence in the BRA assay whereas sCA125 absorption using CHO-K1 parental cells (sCA125 does not bind CHO parental cells, Supplementary Figure 3C and 3D) showed inhibitory activity similar to the original sCA125 preparation (Figure 4D).

To better define how Jurkat-CD16a-farletuzumab interaction was being perturbed by sCA125, we employed farletuzumab and its fragments [(Fab’)_2_ or Fc domains] as competitors. As shown in Figure 4E, sCA125 induced rolling of Jurkat-CD16a was inhibited by full length farletuzumab and the (Fab’)_2_ domain but not by the papain-derived Fc fragment when used in equal molar or fixed (50 μg/mL) concentrations. These results suggest that sCA125 disrupts farletuzumab-CD16a binding via interaction with the (Fab’)_2_ domain. This effect appears to be specific for CA125 since Jurkat-CD16a cells incubated with sFRA antigen in wells coated with farletuzumab did not result in cell rolling (Figure 5H). Full-length farletuzumab itself cannot induce rolling at the concentrations used in the competition studies shown in Figure 4E as the binding of Jurkat-CD16a to well-bound farletuzumab can only be competed with free farletuzumab at concentrations of at least 100-fold (>1 mg/mL). This is likely due to the low affinity of non-antigen bound IgG1 to CD16a receptor as compared to the avidity effect of clustered IgG1 on the well surface [29]. The effect of sCA125 on Fc-receptor-farletuzumab binding appears to be specific for CD16a as the other two Fc-activating receptors, CD32a and CD64a, were not affected by CA125 in the BRA assay as shown in Figure 5D, when expressed in CHO cells along with their co-receptors while recombinant CD32a was affected to a lesser degree than CD16a.

### Interaction of CA125 with (Fab’)_2_ domain perturbs CD16a-Fc-γ receptor binding

Based on the results of the BRA assay (Figure 4E), it is suggested that sCA125 binds to the (Fab’)_2_ region of farletuzumab and perturbs Fc engagement with the CD16a Fc-γ receptor. To confirm this hypothesis, ELISA assays were designed and carried out using recombinant human CD16a-biotin and farletuzumab as well as biotinylated farletuzumab and farletuzumab fragments. Figure 5A shows a representative result of sCA125 binding by farletuzumab and its fragments. Briefly, 15 KU/mL (equivalent to 750 U/well) of sCA125 or human serum albumin (HSA) were used to coat microplates. Wells were washed and probed with equimolar concentrations or 5 μg/mL concentrations of biotinylated-farletuzumab, -(Fab’)_2_ or -Fc fragment. Both full-length farletuzumab and (Fab’)_2_ probes were able to bind to sCA125 in contrast to Fc-biotin or HSA. The farletuzumab-sCA125 binding effect was specific as competition with 100-fold molar excess of unlabeled for letuzumab competed for binding in contrast to HSA (Figure 5B). These results corroborate the competition results in the BRA assay (Figure 4E).

ADCC results using engineered OVCAR-KD lines with suppressed mCA125 levels showed an enhanced activity as compared to OVCAR3 parental cells. To test whether farletuzumab had a differential binding to OVCAR3 vs. OVCAR-KD1, membrane preps were made using subcellular fractionation. Membrane preparations were used to coat microwell plates and then probed using biotinylated-farletuzumab, -HSA or a control antibody that does not bind CA125. Baseline farletuzumab binding to OVCAR-KD1 was similar to its binding to mCA125-negative CHO cells. In contrast, farletuzumab significantly bound to mCA125-positive parental OVCAR3 (Figure 5E). While FRA expression in both lines appeared similar by flow cytometry and as shown in Figure 3E, the membrane fractionation processing used here loses quantifiable amounts of the GPI-anchored FRA whereby the majority is sequestered in the soluble fractions after the last membrane precipitation step as described in the Materials and Methods. Interestingly, this finding suggests that farletuzumab can bind to OVCAR3 membrane via mCA125 independently of FRA antigen and that this binding may have a suppressive impact on immune-mediated cytotoxicity. Similar results were found with other IgG1-type antibodies suggesting this activity is not exclusively inherent to farletuzumab (data not shown).

While both the ELISA data in Figure 5A and the BRA assay data in Figure 4E show sCA125 binds to the (Fab’)_2_ domain of farletuzumab, results of the BRA assay suggest that this interaction affects CD16a engagement with plate-bound farletuzumab Fc domain. To evaluate how this effect may occur, we conducted ELISA assays using unlabeled farletuzumab incubated alone or with sCA125 or HSA and then probed for CD16a-biotin binding. As shown in Figure 5C, when farletuzumab is incubated with sCA125, a ∼46% reduction (ranges from 38-50%) of CD16a-biotin binding occurs as compared to controls (*p* < 0.0002). sCA125 suppression of the CD32a and CD64a Fc receptors was less robust (12% and 1%, respectively), suggesting that CA125 has a more significant impact on IgG1-Fc-γRIIIA engagement than on the other activating Fc receptor family members. This data is supported by the findings using CD16a-, CD32a- and CD64a-transfected CHO cells in the BRA assay whereby sCA125 was able to block adherence of CHO-CD16a cells but not CHO-CD32a or CHO-CD64a (Figure 5D).

Finally, to determine if the effects of sCA125 were specific to farletuzumab, we tested a panel of unrelated (differing variable domain sequences and different antigen binding targets) chimeric (rituximab, cetuximab), humanized (farletuzumab, trastuzumab, MORAb-066, ontuxizumab) or fully human (gimsilumab, MORAb-048) IgG1 mAbs. Two independent IgG1-type antibodies (rituximab and gimsilumab) bound robustly to sCA125 (Figure 5F), and conversely sCA125 inhibited their binding to CD16a-biotin by as much as 50% (data not shown) suggesting sCA125 plays a broad role in the downregulation of IgG1-mediated ADCC via suppression of IgG1-CD16a Fc-γ receptor engagement. Interestingly, rituximab is a chimeric antibody and gimsilumab is a fully human antibody [31]. The similarities of three-dimensional structures of these IgG1s are being studied to determine if common motifs exist that may confer CA125 binding properties.

## DISCUSSION

ADCC is a fundamental immune process utilized by naturally occurring antibodies to protect against infection and aberrant cell propagation. To date, several mAbs have been developed that utilize immune-effector mediated function as part of their pharmacologic mechanism of action (i.e. rituximab, trastuzumab, etc.) [32]. CA125 is one of the best-known TSAs monitored in clinical oncology. Despite its prominent presence in ovarian cancer and other cancer types, little is known of its functional role in tumor biology [33]. Here we show that tumor-produced CA125 has a negative biological effect on NK cell ADCC via direct binding to IgG1 and suppressed engagement with the cell surface CD16a Fc-γ receptor.

Until recently, little clinical evidence has associated CA125 with predictive clinical response to standard or experimental therapy. Its use in clinical testing has mostly been for monitoring [34]. Lack of predictive utility is likely based on the little understanding we have on the biology of this antigen due to its complex biochemical structure and large molecular mass (2.5 - 5.0 MDa), making it difficult to study *in vivo* and *in vitro* [35]. Previous studies have provided evidence that tumor cells expressing mCA125 could potentially block immune cell binding by using its large molecular mass to form a physical barrier for synapse formation [36]. This barrier effect has also been reported in corneal epithelial cells, whereby CA125 interacts with galectin-3 to serve as a barrier against bacterial and viral infection [37]. Its potential effect on immune suppression was furthered by the recent finding that CA125 on mucosal surfaces can bind to immunoglobulins in patients affected with HIV [16]. Others have reported that CA125 may act as an immune suppressor by binding to the Siglec (Sialic acid-binding immunoglobulin-type lectins) NK cell inhibitory receptors Siglec-7 and -9, which in turn downregulate cell surface CD16a-Fc-γ receptor expression. While these functions may be part of a concerted role on how CA125 protects tumor cells from external toxic agents and supports tumor growth, their relevance and impact on clinical outcome need further investigation.

The link between clinical outcome of patients treated with farletuzumab plus SOC and sCA125 levels have provided new insights into how CA125 biology may play a functional role in tumorigenesis, tumor microenvironment immunosuppression, therapeutic response and outcome. As discussed above, sCA125 is routinely used in clinical practice to monitor disease progression and tumor response to therapy. It is also employed to determine prognosis after first line treatment, whereby patients with elevated sCA125 levels have a worse prognosis, even those within the subnormal range [34]. However, the understanding of sCA125 levels to therapeutic outcome and predictive response are limited. The MOR-003-004 Phase 3 clinical trial and subgroup analysis has provided new insights into CA125 systemic levels and response. First, serum CA125 levels do not appear to correlate with tumor lesion size. Patients with large tumor (SLD 200-300 mm) had low and high sCA125 levels similar to that seen in patients that had small tumors (SLD 20-50 mm) suggesting that CA125 levels and clinical effects are not simply a matter of disease bulk. Second, overall clinical outcome in first-relapsed, platinum-sensitive patients treated with SOC chemotherapy was similar in patients with high or low sCA125 levels indicating that sCA125 is not prognostic for carboplatin-taxane based therapy in EOC. Finally, the addition of the immune-mediated experimental agent farletuzumab to SOC showed a significant improvement in both PFS and OS response in patients with low CA125 as compared to placebo control (Figure 1 and Supplementary Figure 1). Since the placebo arm ruled out it being a prognostic effect, these data suggest that an immune-related effect may be a reason for this outcome.

**Figure 6:**
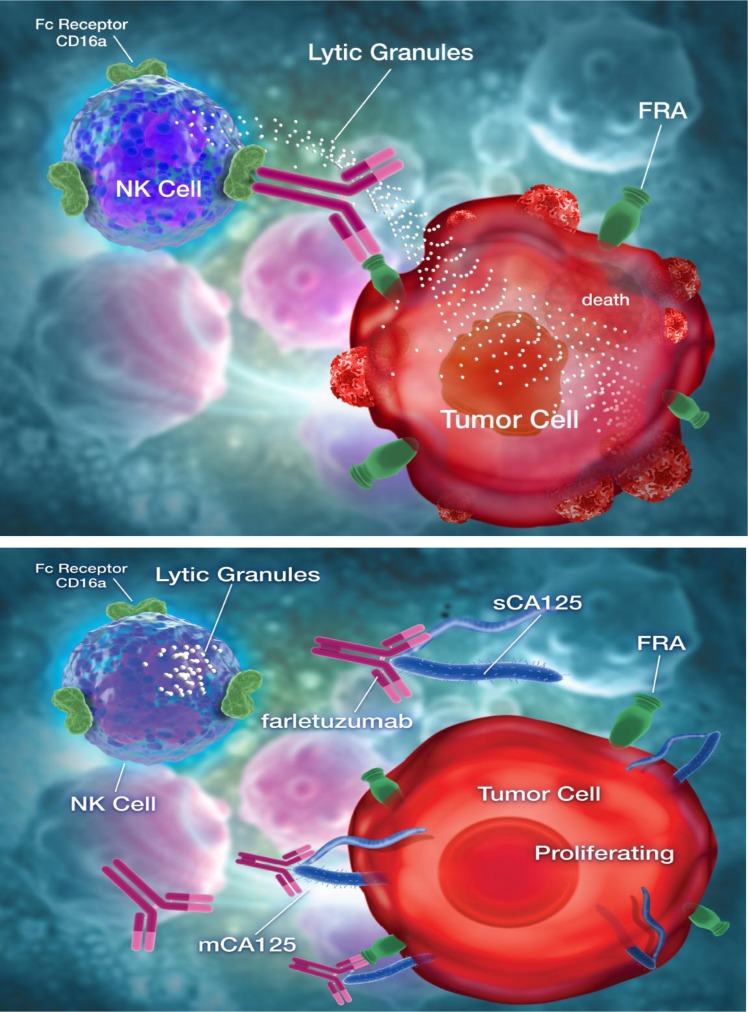
Model depicting farletuzumab-mediated antibody dependent cellular cytotoxicity (ADCC) by NK cells on folate receptor alpha (FRA) expressing tumor cells and ADCC suppression by direct binding of CA125 to antibody Top panel shows farletuzumab binding to its target antigen, folate receptor alpha (FRA), and subsequent binding and activation of NK cells via the CD16a Fc-γ receptor, which in turn leads to release of cytotoxic granules that kills the target cell. Bottom panel shows both soluble and membrane-bound CA125 binding to farletuzumab and suppressing CD16a engagement and ADCC.

Figure 6 depicts the potential mechanism by which CA125 directly binds the (Fab’)_2_ region of farletuzumab and potentially other antibodies and in turn perturbs CD16a engagement. This effect may be a result of allosteric conformational changes to the Fc domain that can occur through V region binding as previously reported [38]. The selectivity of CA125 effect on antibody-CD16a Fc-γ receptor binding may also provide new insights into the functional roles of cells that express CD16a, which is the predominant activating Fc receptor found on NK cells [39]. The ongoing clinical study (NCT02289950) in first-relapsed, platinum-sensitive EOC patients with <3X ULN sCA125 to test the effects of SOC plus farletuzumab vs. placebo on clinical outcome will aid in confirming the effects of CA125 in antibody-mediated response.

CD32a and CD64a receptors represent the other activating Fc receptors in the human immune system. Both CD32a (FcγRIIA) and CD64a (FcγRI) are primarily expressed by monocytes, macrophages, granulocytes and dendritic cells (DCs) [40]. CD16a/FcγRIIIA is primarily expressed on NK cells, DCs and a subset of monocytes. Both CD32a and CD16a represent low affinity IgG1 receptors while CD64a represents a high affinity receptor. As shown in Figure 5C and 5D, CD16a appears to be the most sensitive to CA125-IgG1 binding and CD32a is affected to a lesser degree. These data suggest that CA125 may have an inhibitory role that not only affects ADCC via CD16a on NK cells but also myeloid-mediated immune responses via CD32a. CD64a was unaffected by CA125, which may be due to its higher affinity for IgG1 or its differentiated structure and function [41]. Further analysis is ongoing to define the CA125 antibody binding motif, its effect on antibody Fc structure and impact on the immune cells expressing those Fc receptors affected by its binding to aid in further elucidating our findings.

## EXPERIMENTAL PROCEDURES

### Quantitation of TSAs from patient serum

Pre-treatment serum samples obtained from subjects in the Phase 3 trial were evaluated for CA125, CA19-9, CA15-3, AFP (α1-fetoprotein) and CEA (carcinoembryonic antigen) levels using the Roche COBAS 6000 platform. Testing was performed in a CLIA accredited laboratory at Aspira Labs, Austin, TX.

All assays employ an electrochemiluminescence immunoassay format. Assays are single-step sandwich assay incorporating a streptavidin-coated microparticle, a biotinylated-capture antibody and a ruthenylated-detection antibody.

All protocol and informed consent documentation received institutional review board approval in accordance with the Declaration of Helsinki. Detailed study information and written informed consent was provided to each patient before any study-specific screening.

### Statistical analysis of TSAs and clinical response

Cut-point analysis of baseline TSAs in the Phase 3 population (*entry set*) and *post hoc set* were performed using maximal chi-square methodology [42] and stratified log-rank tests were used to compare PFS and OS among treatment arms. A stratified proportional hazard Cox model was applied to calculate HRs for PFS and OS by comparing farletuzumab to placebo treated groups with high vs. low TSA levels. All reported p values are two-sided unless otherwise specified. Kaplan-Meier curves were generated on median PFS and p values from a log-rank test stratified by various covariates. The HR (farletuzumab:placebo) is estimated based on a Cox proportional hazards model stratified by the same factors as those for log rank test.

### Cell culture

The human ovarian cancer cell lines OVCAR3, CAOV3, Jurkat and CHO-K1 were all obtained from ATCC. IGROV1 was obtained from NCI. All were grown in complete RPMI or DMEM medium as suggested by the vendor. All cells were maintained at 37°C/5% CO_2_.

### sCA125 preparations

sCA125 preparations were obtained from primary patient ascites, pooled and purified by filtration, affinity and size exclusion at Morphotek or independent vendors Lee Biosolutions (Maryland Heights, MO) or Antibodies-online (Atlanta, GA). Purified preparations were done as described in the Supplementary Methods.

### Generation of recombinant Jurkat cell lines

Jurkat-CD16a lines were generated by electroporation using equivalent amounts of plasmids encoding open reading frames of human CD16a and FCεR (CD16 receptor complex) and selection with 400 μg/ML G418 and 2.5 μg/mL blasticidin. Surface expression of CD16a was confirmed by flow cytometry.

### Lipofectamine transfection of CHO cells

CMV promoter-driven expression plasmids were used to transfect cells using lipofectamine 2000 as recommended by the vendor (Thermo-Fisher). Cells transfected with FRA and mesothelin vectors were selected in 400 μg/mL zeocin. Cells transfected with CD16a, CD32a or CD64a Fc receptors were selected using 2.5 μg/mL blasticidin (FceR) and 400 μg/ML G418 (CD16a, CD32a and CD64a). Surface expression of each line was confirmed by flow cytometry.

### shRNA knockdown of mCA125 expression in cancer cells

CA125 knockdown was performed using shRNA Mission Lentiviral particles as recommended by the vendor (Sigma-Aldrich, St. Louis, MO). The catalog numbers are in the Supplementary Methods. Vectors targeting different regions of the mRNA were used for cell transductions and stable integration. Single cell subclones were expanded and screened for CA125 knockdown and FRA expression by flow cytometry with the OC125 antibody (Abcam, Cambridge, UK) or 26B3 [43], respectively, followed by staining with FITC-conjugated secondary antibody. Clonal cell lines were monitored over multiple passages for continued suppression. Effective shRNA constructs used in experiments were TRCN0000262688 (KD1: 5′- CCGGTCACATCTCCAATGGTTATTAC TCGAGTAATAA-CCATTGGAGATGTGATTTTTG-3′) and TRCN0000262686 (KD3: 5′- CCGGT-GCCGTTCACCCTCAACTTTACTCGA GTAAAGTTGAGGGTGAACGGCATTTTTG-3′).

### Antibody-dependent cellular cytotoxicity

ADCC assays were conducted using primary cells or Jurkat-Luc (Promega) effector cells as described in the Supplementary Methods. Percent ADCC inhibition was calculated as 1 - (sCA125-treated / untreated) x 100.

For assays using human PBMC effector cells, effector and target cells were plated at 8:1 effector:target cell ratio +/− 20,000 U/mL sCA125.

For assays using NK cells, cells were isolated from human PBMCs using the EasySep™ Human NK Cell Enrichment Kit (StemCell Technologies, Cambridge, MA) as per the manual and target cells were plated at 8:1 effector:target cell ratio with increasing concentrations of sCA125.

### sCA125 antibody binding assays

Various assays were carried out and provided in detail in the Supplementary Methods. These include: effects on CD16a and Siglec expression, effector and target cell viability in presence of sCA125, binding of CA125 to effector or target cells and effect of farletuzumab binding to target cell in presence of sCA125.

### Biological rolling assay

U-bottom microplates (untreated) were coated overnight with antibody and fragments, fibronectin or BSA with 100 mL of 10 μg/mL protein in 0.2 M bicarbonate buffer, pH 9.4 at 4°C. Wells were washed and blocked with 200 mL PBS/1% BSA for 1 hr at room temperature. 2.5×10^4^ Jurkat or CHO cell transfectants expressing CD16a, 32a or 64a were added with or without sCA125 to wells in a total volume of 100 μL ADCC assay buffer (Promega). Microplates were placed at 37°C/5% CO_2_ for 5 hr then imaged using an EVOS imaging system (Thermo-Fisher, Waltham, MA). Cell cluster diameter was quantitated by A_405_ using a SpectraMax M5 (Molecular Devices, Downingtown, PA). sCA125 competition studies were done using 50 μg/mL of antibody or antibody fragment. sCA125-mesothelin absorption is described in Supplementary Methods.

### ELISA analysis of antibody-CA125 binding

ELISAs were carried out as previously described [44]. Human CD16a-, CD32a- and CD64a-biotin probes were purchased from Sino Biological Inc. Biotinylated CA125 or HSA were generated as previously described [45]. Antibodies and fragments were biotinylated using sulfo-tag conjugation as described (Meso Scale Diagnostics Inc). Subcellular fractionation was carried out as recommended by the vendor (Abcam, Cambridge, UK) and in detail in Supplementary Methods. All reactions were done in at least triplicate.

### Statistical analysis on laboratory experiments

Mean values were compared using an unpaired Student's two-tailed t test.

## SUPPLEMENTARY MATERIALS FIGURES



## Abbreviations

(ADCC): Antibody dependent cellular cytotoxicity
(EOC): epithelial ovarian cancer
(FRA): folate receptor alpha
(HR): hazard ration
(<3X ULN): no greater than three times the upper limit of normal
(OS): overall survival
(PFS): progression free survival
(SLD): sum of the longest diameter
(SOC): standard of care
(sCA125): soluble CA125
(mCA125): membrane bound CA125
