# Supplementary material for: Tumor antigen CA125 suppresses antibody-dependent cellular cytotoxicity (ADCC) via direct antibody binding and suppressed Fc-γ receptor engagement
Source: Oncotarget. 2017 Jul 7;8(32):52045–60. doi: 10.18632/oncotarget.19090 (PMC5581011; doi:10.18632/oncotarget.19090)
Supplement: Supplementary file 1 [file oncotarget-08-52045-s001.pdf]

Tumor antigen CA125 suppresses antibody-dependent cellular cytotoxicity (ADCC) via direct antibody binding and suppressed Fc-γ receptor engagement

SUPPLEMENTRAY MATERIALS

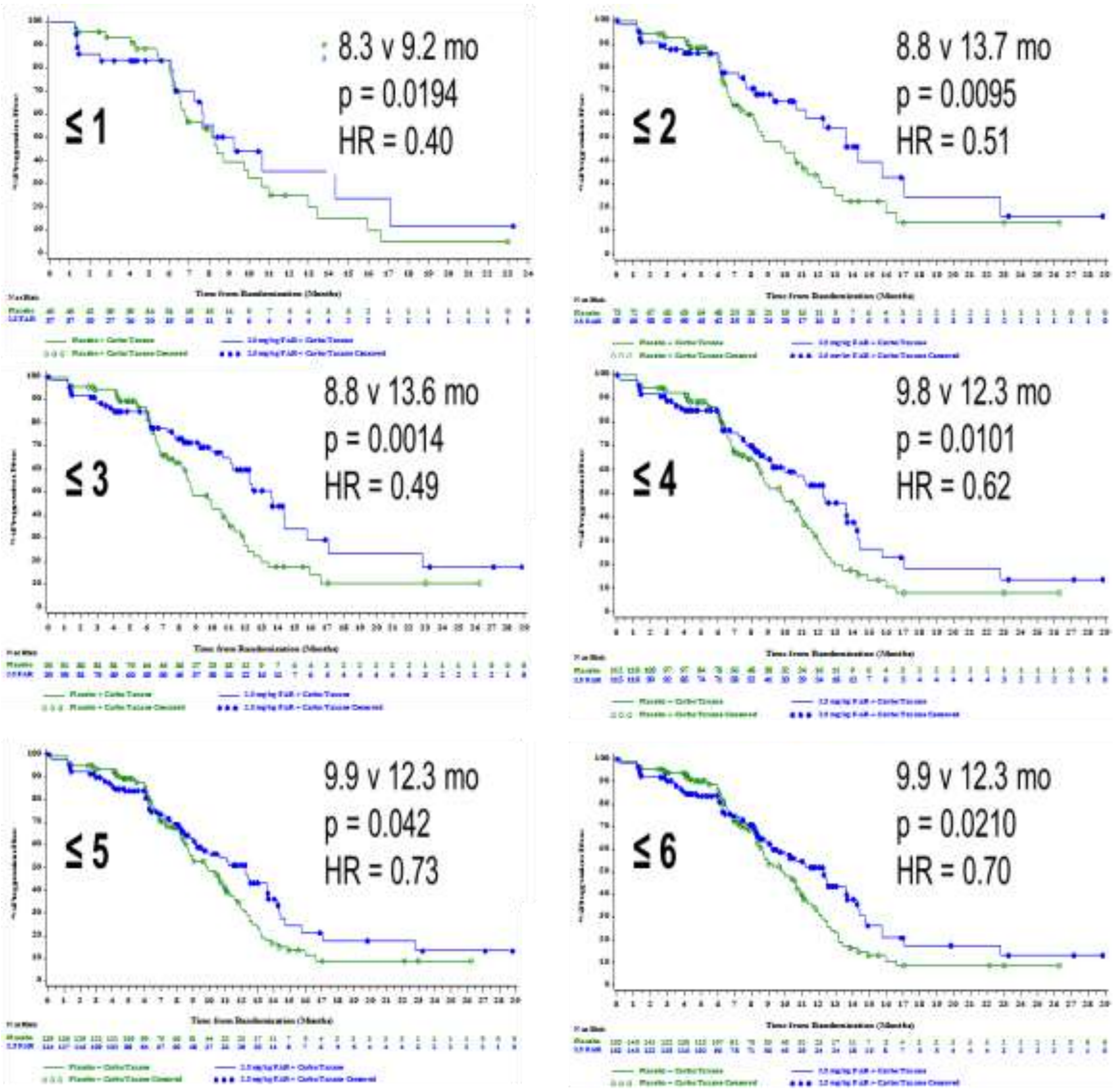

Supplementary Figure 1. Kaplan Meier curves measuring median PFS response in subsets of patients with varying levels of baseline serum CA125 (indicated by the bold number in each graph) treated with chemotherapy (carboplatin + taxane) plus placebo (green line) or 2.5 mg/kg farletuzumab (blue line). As shown and consistent with the

bubble graph in figure 1A, patients with CA125 less than 3X the upper limit of normal have a significant clinical response when treated with farletuzumab plus SOC as compared to placebo plus SOC. In general, an approximately linear relationship can be observed where as CA125 rises, the observed clinical effect diminishes. Of 23 covariates evaluated in cox regression analysis, CA125 was the only factor with an observed effect predictive of farletuzumab response.

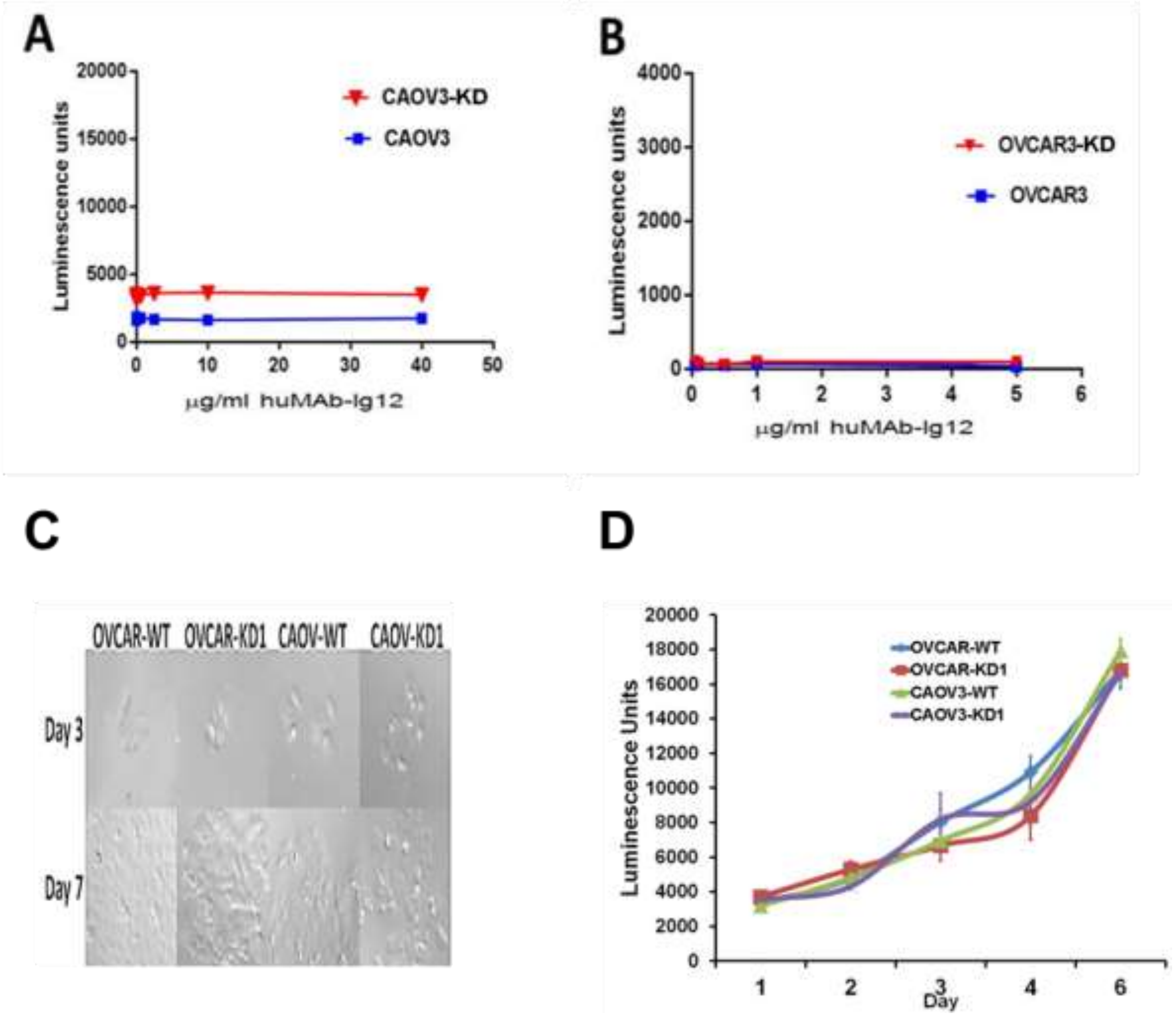

Supplemental Figure 2. Jurkat-Luc ADCC signaling is antigen specific. **A)** CAOV3 parental and CAOV3-KD cells were incubated with Jurkat-Luc cells plus a humanized antibody huMAb-Ig12 whose antigen is not expressed on CAOV3 cells. As shown no signal is observed for either cell line demonstrating ADCC specificity of Jurkat-Luc and farletuzumab on CAOV3 cells. **B)** OVCAR3 parental and OVCAR3-KD cells were incubated with Jurkat-Luc cells plus a humanized antibody whose antigen is not expressed on OVCAR3 cells. As shown no signal is observed for either cell line

demonstrating ADCC specificity of Jurkat-Luc and farletuzumab on OVCAR3 cells. Note: huMAb-Ig12 does not bind sCA125. **C)** Cell morphology of CA125 knockdown (KD) subclones is identical to parental lines. **D)** Growth rates of CA125 KD subclones are identical to parental lines.

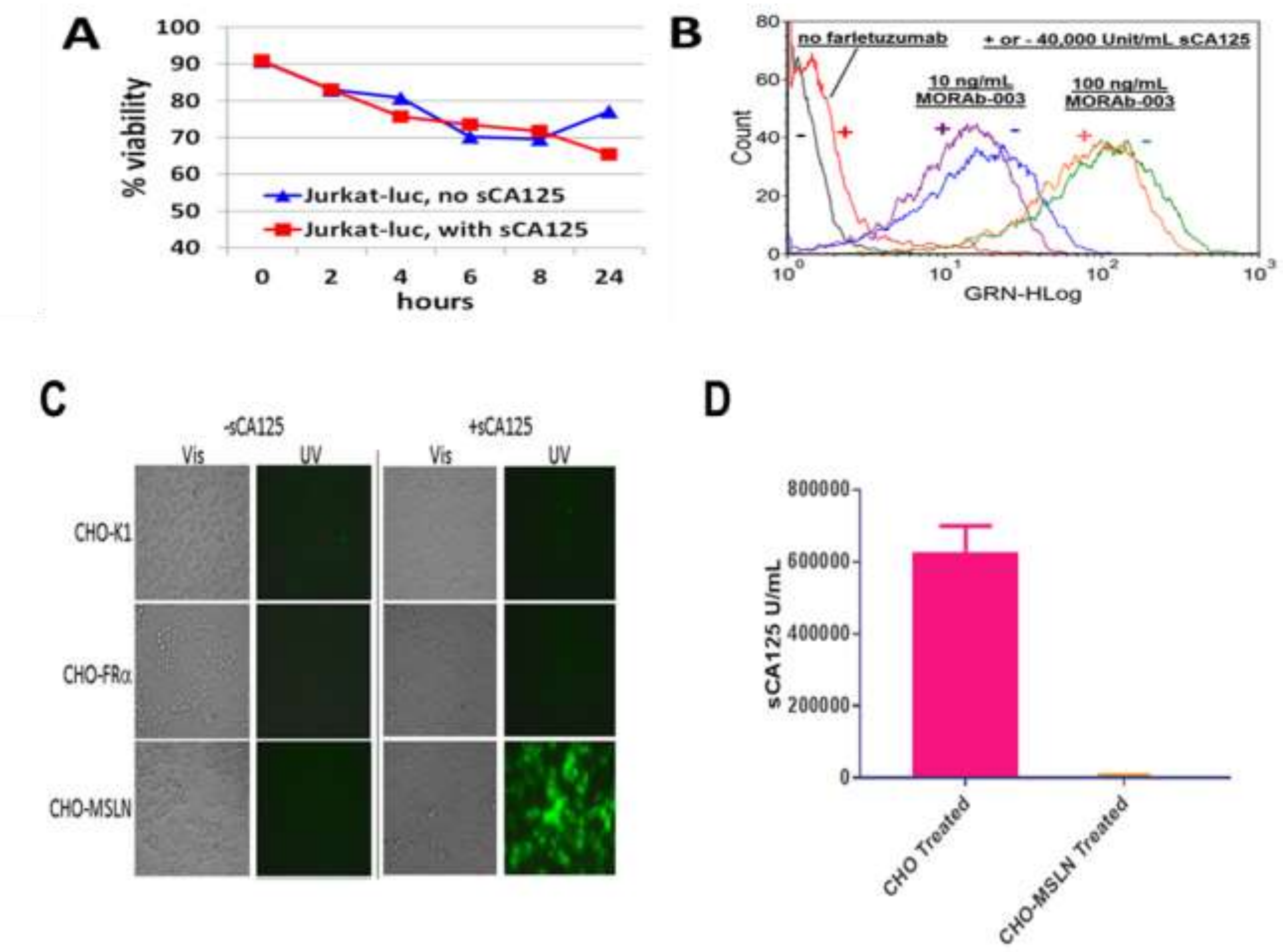

Supplemental Figure 3. sCA125 has no effect on effector or target cells alone. **A)** Jurkat-Luc cells were incubated with sCA125 (red line) and tested for cell viability as compared to cells not exposed to sCA125 (blue line). As shown, sCA125 does not reduce viability of Jurkat-Luc cells. **B)** IGROV1 cells were exposed to sCA125 (purple and orange lines) and tested for FRA cell surface expression as compared to IGROV1 untreated (blue and green lines) using two concentrations of farletuzumab (MORAb-003) antibody by flow cytometry. sCA125 does not affect FRA cell surface expression nor affect decreased farletuzumab binding on IGROV1 cells. **C)** sCA125 binds CHO-MSLN cells but not CHO or CHO-FRA cells. CHO and CHO-FRA cells were tested for sCA125 direct binding. CHO-MSLN was used as a positive control to demonstrate CA125 binding ability within this assay format. MSLN (mesothelin) is a cell surface protein known to bind CA125 at high affinity. Vis, bright field; UV, field under UV light. **D)** Analysis of CA125 supernatant after

incubation with CHO parental or CHO-mesothelin (CHO-MSLN) expressing cells. Post-incubation sCA125 supernatant was analyzed for CA125 via an anti-CA125 immunoassay and shown to be depleted from supernatant after incubation with CHO-MSLN cells but not CHO parental.

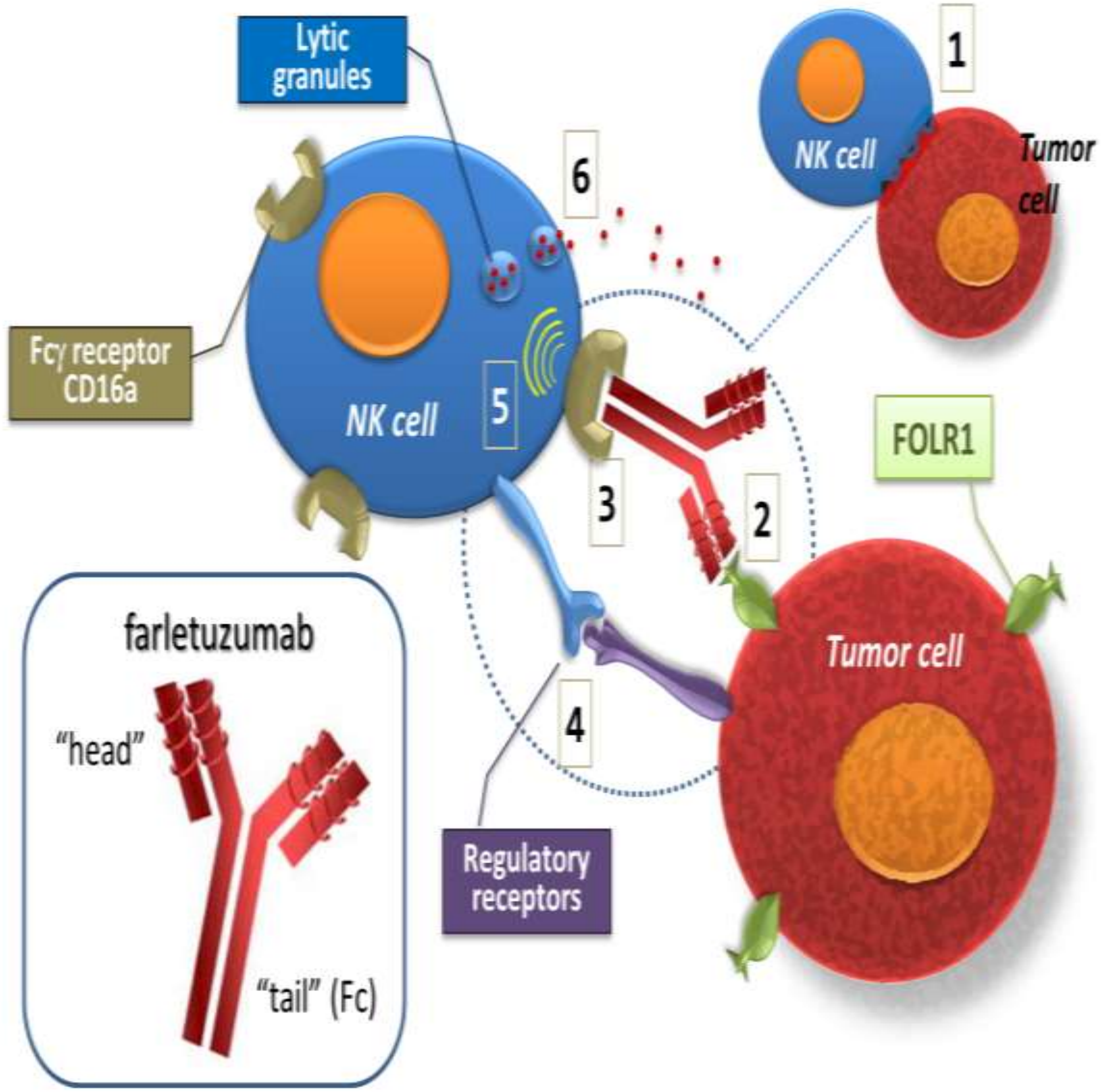

Supplemental Figure 4 . Six steps culminating in ADCC
1 – CD16a-effector cell (i.e. NK cell) engages the tumor cell through the synapse, an area of tight cellular contact. The synapse region is enlarged in the middle section of the figure (dashed circle). **2** – The "head" of farletuzumab or an IgG1 antibody binds FRA or cell surface target receptor and recruits the effector cell via its "tail" (the Fc domain). **3** – The

NK cell recognizes farletuzumab/antibody via CD16a binding. **4** – The engagement of regulatory receptors, as well as proteins including adhesion molecules, completes the synapse formation. **5** – As a result, a cellular signal emanating from CD16a leads to lytic granules release. **6** – Lytic granules pour out of the NK cell and ultimately cause cytotoxicity.

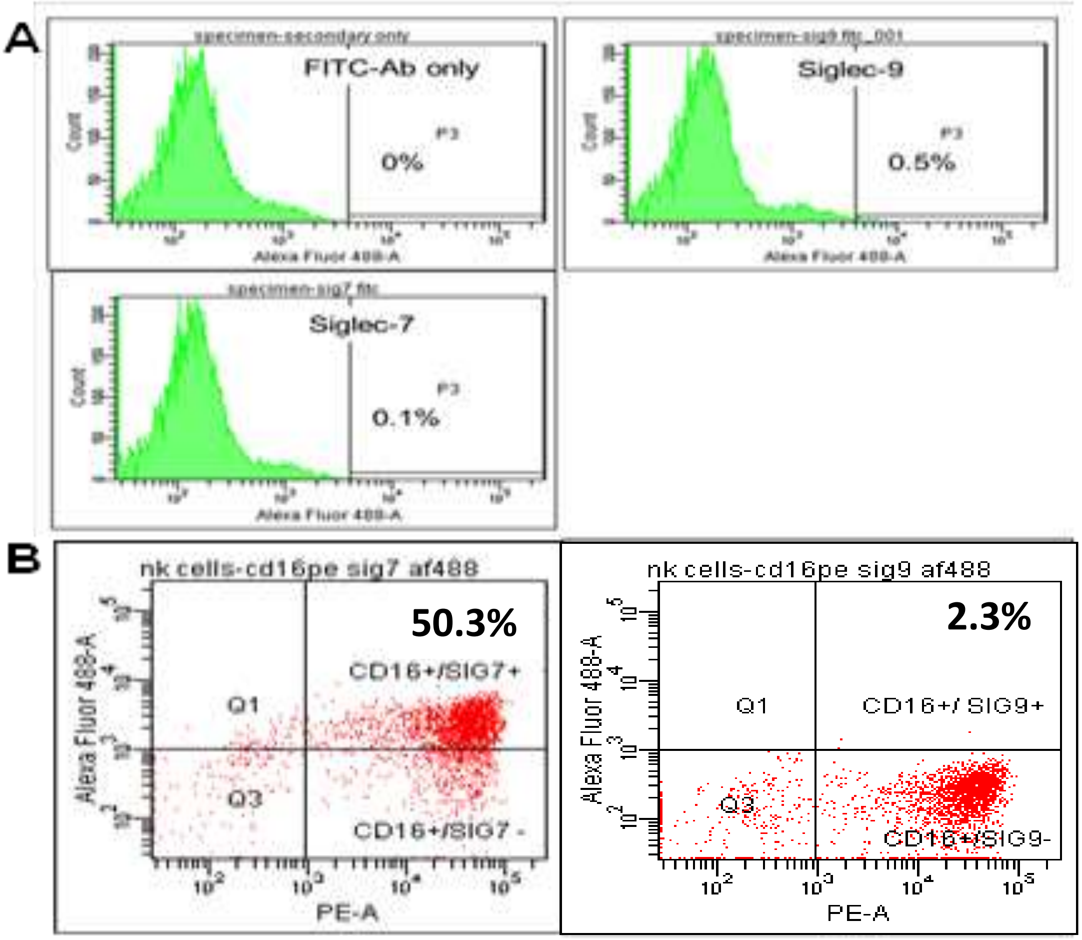

Supplemental Figure 5. A) Jurkat-Luc cells were analyzed via flow cytometry for expression of the NK inhibitory receptors Siglec-7 and Siglec-9. As shown, Jurkat-Luc cells expressed little to no cell surface expression of either receptor. **B)** Primary human $CD16^+$ NK cells were analyzed via flow cytometry for expression of the NK inhibitory receptors Siglec-7 and Siglec-9. As shown ~50.3% of $CD16^+$ NK cells express Siglec-7 and ~2.3% of $CD16^+$ NK cells express Siglec-9.

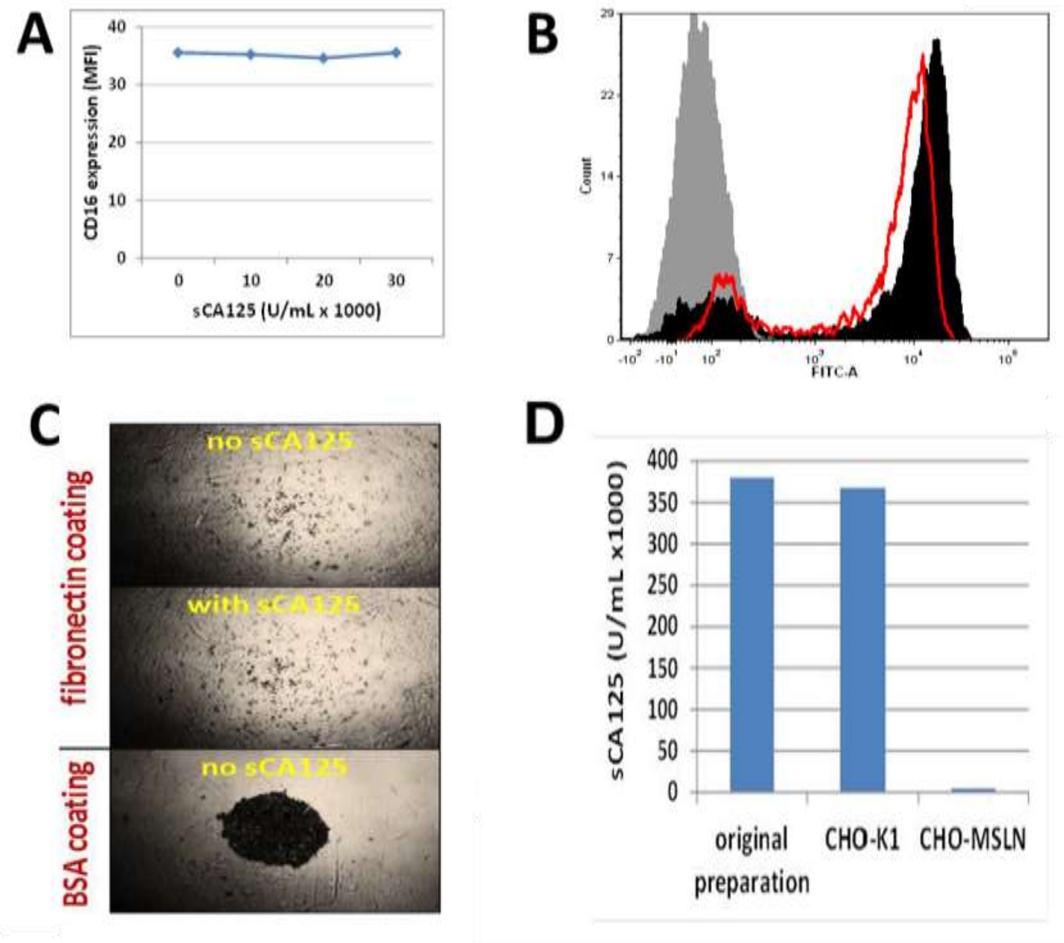

Supplemental Figure 6. Validating components of the biological rolling assay (BRA assay). To test for potential inhibitory effects of sCA125 on CD16a expression by Jurkat-CD16a or nonspecific effects on Jurkat-CD16a binding to well surfaces, Jurkat-CD16a cells were incubated with sCA125 and tested for: **A)** effects on CD16a. Flow cytometry analysis of CD16a expression on Jurkat-CD16a cells treated with increasing concentrations of sCA125. As shown sCA125 has no effect on CD16a cell surface expression; **B)** Primary NK cells were analyzed via flow cytometry for expression of CD16a when cells were treated with PBS or sCA125 for 5 hours at 37ºC in 5%CO_2. Gray peak indicates unstained cells, black peak indicates CD16 staining of untreated (PBS) cells, red overlay indicates NK cells treated with sCA125. **C)** sCA125-CD16a specificity. Microwell surface binding of Jurkat-CD16a to fibronectin shows that it can support cell adherence to well wall surface (top panel) and sCA125 treatment does not inhibit fibronectin-dependent adherence (middle panel); BSA-coated wells were used as negative control as Jurkat nor Jurkat-CD16a bind BSA. As shown, Jurkat-CD16 cells do not adhere to BSA-coated wells and therefore roll to the bottom of the well (bottom panel). **D)** CHO-MSLN cells are robust binders to sCA125. sCA125 ELISA showing depletion of sCA125 from its buffer after adsorption to CHO-MSLN cells. sCA125 preps were also exposed to CHO-K1 parental cells and shown not to remove any sCA125 from the buffer as compared to starting material shown in the first column.

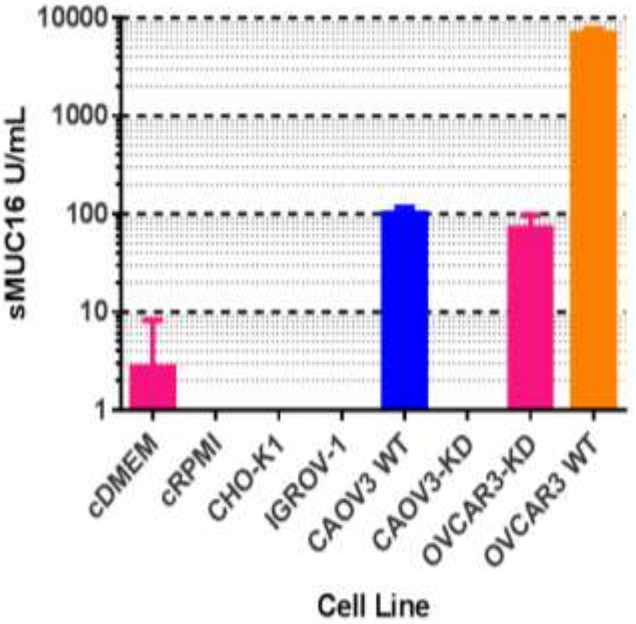

Supplemental Figure 7. Levels of shed CA125 (sCA125) by different cell lines. Briefly, $2x10^5$ cells were cultured for seven days in 2 mLs of cDMEM (CAOV3 lines) or cRPMI (all other lines) in 6 well plates. Supernatants were harvested and sCA125 quantitated by an immuno-based FRET assay using europium cryptate and d2-labeled anti-CA125 antibodies (Lee BioSciences 151-30) against a standard curve of 0-500 U/mL sCA125. cDMEM and cRPMI are cell-free growth media used to measure background levels. As shown OVCAR3 produces ~100-fold more sCA125 than CAOV3 or its isogenic knockdown line OVCAR3-KD.CAOV3-KD appears to have completely lost production of sCA125 as compared to its isogenic parental line.

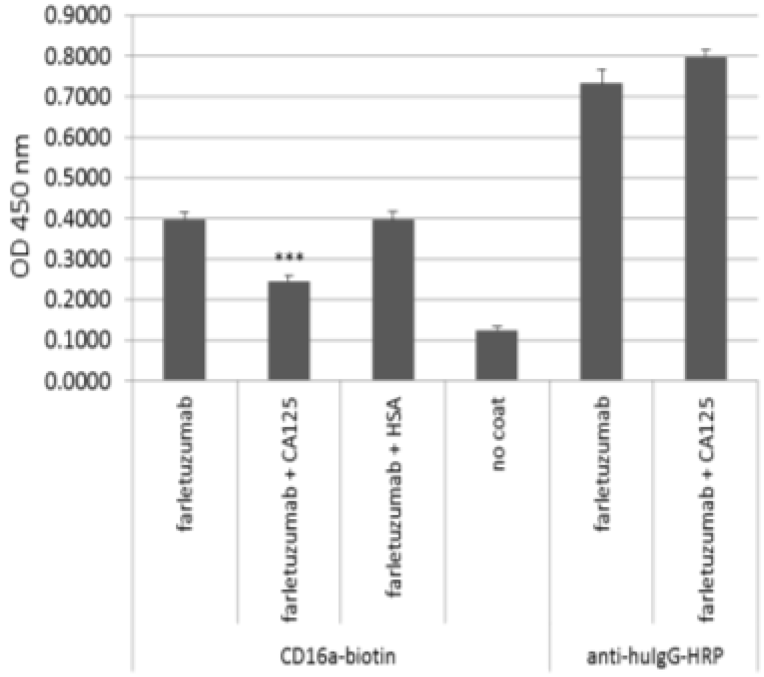

Supplemental Figure 8. Farletuzumab was incubated alone or with sCA125 or HSA overnight and probed with human CD16a-biotin. Shown is the reduced effect of CD16a binding to farletuzumab incubated with sCA125. Reduced CD16a binding is not a result of less farletuzumab as probing wells with anti-human IgG-HRP (anti-huIgG-HRP lanes) show that similar amounts of farletuzumab are present in CA125 and control wells.

Supplemental Methods and Procedures

Cell culture
The human ovarian cancer cell lines OVCAR3 (ATCC, Rockville, MD) and IGROV1 (NCI, Frederick, MD) were grown in complete RPMI 1640 (cRPMI, Thermo-Fisher, Waltham, MA) supplemented with 10% FBS (Thermo-Fisher), 2 mM L-glutamine (Thermo-Fisher), 100 U/mL penicillin, 100 μg/mL streptomycin. Cells were maintained at 37°C in humidified 5% CO_2 incubators. CAOV3 (ATCC) and CHO (CHO-K1, ATCC) derived cell lines were grown in complete DMEM (cDMEM, Thermo-Fisher, Waltham, MA) supplemented with 10% FBS (Thermo-Fisher), 2mM L-glutamine (Thermo-Fisher), 100 U/mL penicillin, 100 μg/mL streptomycin. Cells were maintained at 37°C in humidified 5% CO_2 incubators.

Antibody-dependent cellular cytotoxicity
ADCC assays were conducted using primary cells or Jurkat-Luc effector cells, the latter of which is part of the ADCC Reporter Bioassay Core Kit Protocol and assayed as recommended by the vendor (Promega). Antibody, effector and target cells were incubated with varying amounts of sCA125 in ADCC assay buffer. Percent ADCC inhibition was calculated as 1 - (sCA125-treated / untreated) x 100.

For assays using human PBMCs as effector cells, target cells (5 x 10^2/well/40 μL cDMEM) were seeded in U-bottom microplates along with IL-2-stimulated human PBMCs (4 x 10^3/well/40μL cDMEM) at 8:1 effector:target cell ratio. Antibodies were added with or without 20,000 U/mL sCA125 and incubated at 37°C/5% CO_2 for six hours. Cells were then washed, transferred to a 96 well flat bottom microplates, grown for 7 days at 37°C / 5% CO_2 and quantified using CellTiter-Glo® (Promega) as recommended by the vendor to compute live cells. Percent cytotoxicity was computed as 1 - (treated / untreated) x 100.

For NK cell ADCC, NK effector cells were isolated from human PBMCs using the EasySep™ Human NK Cell Enrichment Kit (StemCell Technologies, Cambridge, MA) as per the manual. Antibody/CA125 mixes were added to wells along with 2 x 10^4 NK cells (2:1 E:T ratio). Varying CA125 concentrations (0 to 50 KU/mL) were added with or without 6 μg/mL of antibody. Cultures were incubated for 5 hours at 37°C/5% CO_2, then aspirated and washed to remove non-adherent effector cells and assayed for viable cells using CellTiter-Glo®. Cytotoxicity was calculated as 1-(Ab+sCA125 treatment)/(effector+target spontaneous killing) x 100%. Inhibition of killing by CA125 was normalized to killing with antibody in the absence of CA125.

shRNA knockdown of mCA125 expression in cancer cells
CA125 knockdown by shRNA was performed using shRNA Mission Lentiviral particles purchased from (Sigma-Aldrich, St. Louis, MO). The seven independent shRNA lentiviral constructs tested were catalog numbers: TRCN0000262688, TRCN0000262687, TRCN0000262686, TRCN0000262685, TRCN0000262684, TRCN0000180917, TRCN0000179369. The most robust knockdown shRNA constructs used in our experiments were catalog numbers TRCN0000262688 (KD1) and TRCN0000262686 (KD3).

Characterization of CA125-KD lines

Parental and knockdown growth rates were observed by seeding sterile 96 well tissue culture plates with 500 cells per well in 100μL cRPMI across columns in duplicate. Every 24 hours, 75μL cell titer glow (Promega) were added to a column of cells and transferred to an white opaque 96 well plate and luminescence quantified as a readout for viable cell numbers. Wells after one week of growth were also imaged at 40x to show similar cellular phenotypes between parental and knockdown lines.

Assays to validate ADCC suppression is through direct antibody binding by sCA125

-Analysis of surface CD16a expression in presence of CA125

Increasing concentrations of sCA125 were added to Jurkat/Jurkat-CD16a and CHO/CHO-CD16a transfected cells for 5 hrs in growth media. Cells were stained for 30 min on ice with FITC-labeled anti-hCD16 antibody (BD Biogen, San Jose, CA), washed with FACS buffer (1%FBS/PBS) and analyzed for CD16 expression and viability by flow cytometry using Via-Probe (BD Biosciences) 2-color analysis using a FACSAria (Beckton-Dickinson)..

-Analysis of Siglec expression

Jurkat cells were stained for 30 min on ice with 10 μg/mL goat anti-Siglec-7 or Siglec-9 (R&D Systems) in FACS buffer. Cells were washed twice with FACS buffer followed by staining with 10 μg/mL AF488-conjugated donkey anti-goat secondary antibody (Thermo-Fisher) for 30 min on ice in FACS buffer. Cells were washed and analyzed by flow cytometry for Siglec staining using a FACSAria. Isolated NK cells were stained with 10 μg/mL PE-anti-CD16 antibody in conjunction with FITC-labeled anti-Siglec-7 or -9 antibodies as above..

-Analysis of sCA125 binding on target or Jurkat cells

Cells (6×10^4/well) were seeded in flat bottom microplates in 100 μL cRPMI or cDMEM and grown overnight at $37^oC/5\% CO_2$. The following day, 30,000 U/mL of sCA125 was added to culture and incubated for two hours at $37^oC/5\% CO_2$. Media was aspirated and cells were washed with FACS buffer. Anti-CA125 antibody OC125 (1 μL/well) was added for 30 min on ice, then washed twice and 1 μL/well of goat anti-mouse-FITC secondary antibody (Rockland) was added and incubated for 30 min on ice. Cells were washed and imaged by confocal microscopy under visible light (20 msec) and UV light (5 sec) at 200 x magnification.

-PBMC, NK cells and Jurkat-Luc cell viability

Frozen vials of Jurkat-Luc and donor PBMCs were thawed in a 37^oC water bath. NK cells were isolated from PBMCs as above. Cells were diluted to 2×10^6 cells/mL in warm cRPMI and each cell type split into 15 mL tubes. One of each cell type was mixed with sCA125 (Lee Biosolutions) at a final concentration of 30,000 U/mL. Tubes were placed at $37^oC/5\% CO_2$ and 0.1 mL samples mixed with 0.9 mL cRPMI (1:10 dilution) and viability assessed using a Vi-CELL cell counter (Beckman Coulter, Sharon Hill, PA).

-Farletuzumab binding on target cells in the presence of sCA125

1 x 10^6 cells of IGROV1 cells in FACS buffer were incubated on ice with sCA125 for 30 minutes. Farletuzumab (10 or 100 ng/mL) was then added for 1 hr then cells were washed and goat anti-human-Alexa Fluor® 488 secondary antibody (Thermo Fisher Scientific Inc.) was added at 1 μg/mL for 20 minutes. Cells were then washed, fixed for 10 min in 10% formalin and fluorescence intensity measured by flow cytometry.

CA125 purification from pooled patient ascites. Patient ascites are pooled and then buffered to final concentration of 3.3 mM Tris, 1.6 mM EDTA, pH7.5. Samples are then centrifuged at 18,000x g for 15 min at RT and filtered twice using 0.8 μm and then 0.2 μm filters, respectively. Samples were then fractionated by FPLC using superose 6 10/300 GL and high molecular weight fractions were concentrated using 50K MW exclusion filters. Next a mesothelin agarose column was used. The mesothelin protein has been shown to be a high-affinity binding protein with a KD of 5 nM as reported by Gubbels (15) and referenced in the text. Samples were then added to mesothelin-agarose affinity columns, washed 10 times with Dulbecco's PBS (DPBS), 2 times with 20 mM Tris, 150 mM NaCl, pH 7.5 and eluted from the column using 50 mM MOPS, 3 M MgCl2, pH 7.0 and equilibrated in 20 mM Tris, 150 mM NaCl, pH 7.5. Eluted material was desalted 2 times using HiTrap desalting columns with DPBS as running buffer and concentrated using 30K MW cut off centrifugal filters. Samples were fractionated one last time via FPLC using superose 6 10/300 GL and the remaining high molecular weight fraction was concentrated using 30K MW exclusion filters. The figure below shows the homogeneity of the high molecular weight material and the corresponding gel is a 4-12% Bis Tris SDS PAGE gel showing typical starting material (lane 1) and final purified product from independent isolations (lanes 2 and 3). As CA125 is a highly glycosylated protein, standard dyes do not stain it well and is recommended that it be detected using periodic acid-Schiff (PAS) as recommended by the manufacturer (Thermo Scientific). Below is a typical chromatograph of preparations from pooled human ascites after 2 rounds of high molecular weight exclusion (lane 1) and chromatographic purification via multiple rounds of FPLC and affinity isolation (lanes 2 and 3). Protein is confirmed to be CA125 as by immunologic analysis.

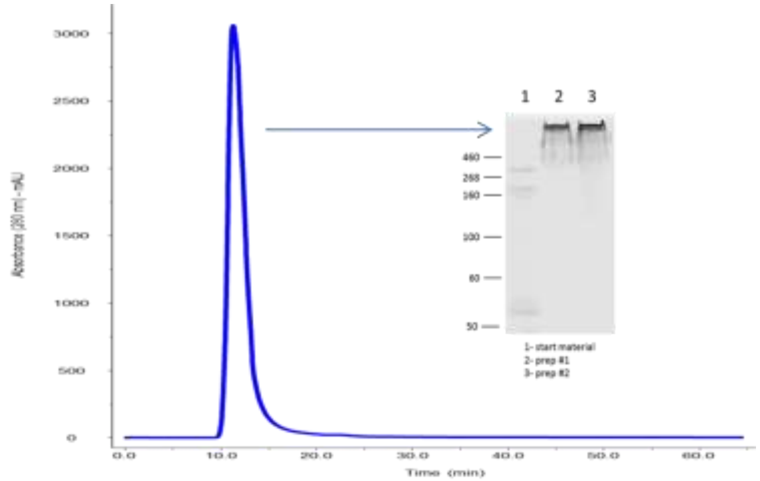

sCA125 absorption and quantitation

CHO or CHO-MSLN cells (5 x 10^6) were washed with cold PBS, pelleted, and resuspended in 100 μL of sCA125 solution and incubated on ice for 90 min. Cells were pelleted at 1,000 RPM for 3 minutes at 4°C, and supernatant collected. sCA125 concentration in supernatants were quantitated by FRET analysis against the sCA125 standard starting material. Briefly, sCA125 standard and samples were serially diluted 1:1 in cRPMI. FRET analysis using Europium cryptate and d2-labelled anti-CA125 antibodies (Lee Biosolutions) was performed in opaque white 384 well microplates (Greiner) in the presence of diluted samples and control wells for 3.5 hours at room temperature. FRET values were measured using a Wall-E microplate reader (Paradigm).

ELISA analysis of antibody-CA125 binding

ELISAs were carried out by diluting corresponding antigens (CA125, antibody, HSA) to final concentration in 0.05M carbonate buffer, pH9.5 or phosphate buffered saline and immobilized in polystyrene plates overnight at 4°C. Plates were washed with 0.05M PBS, pH7.2 (PBS) and blocked with 5% BSA in PBS for 1 hr at room temperature. Plates were rinsed then probed with biotinylated agents diluted in PBS plus 0.5% BSA for 1 hr at room temperature. Plates were rinsed three times, washed for 10 min at room temperature 0.05M PBS, pH7.2 then probed with streptavidin-horse radish peroxidase (Jackson Immuno Research) diluted 333 ng/mL in PBS plus 0.5% BSA for 1 hr at room temperature. Plates were washed as above and TMB substrate solution was added for 15 min. Reactions were stopped with 0.16M H_2SO_4 and absorbance read at 450 nM using a SpectraMax M5 plate reader.

Cellular subfractionation

Cells were grown to 90% confluence and removed from plate using Cell Dissociation Buffer, enzyme-free, PBS (Gibco). Cells were washed in PBS and resuspended in 500 µL of ice cold fractionation buffer 250mM sucrose, 20mM HEPES, pH7.4, 10mM KCl, 2 mM MgCl2, 1 mM EDTA, 1 mM EGTA and 1X Halt Protease Inhibitor (Thermo Scientific). Cell suspension was sheared through a 25 gauge needle 10 times using a 1 mL syringe and incubated on ice 20 min. Supernatants were centrifuged 3,000 rpm at 4°C for 5 min. Supernatant containing cytoplasm, membrane and mitochondria were next centrifuged 8,000rpm in a microfuge at 4°C for 5 min. Pellet supernatant containing cytoplasm and membrane were centrifuged at 50,000g for 2 hrs at 4°C using a high speed Beckman centrifuge. The membrane pellet was washed using 400 µL of fractionation buffer and recentrifuged for 1.5 hrs at 4°C. Membrane pellet was then resuspended in 500 μls of ice cold TBS buffer (50 mM Tris-Cl, pH 7.6; 150 mM NaCl) plus 1X Halt Protease Inhibitor. Preps were quantitated by Coomassie blue and ELISA.
